# Quantification of the Direct Solar Impact on Some Components of the Hydro-Climatic System

**DOI:** 10.3390/e23060691

**Published:** 2021-05-31

**Authors:** Constantin Mares, Ileana Mares, Venera Dobrica, Crisan Demetrescu

**Affiliations:** Institute of Geodynamics of the Romanian Academy, R-020032 Bucharest, Romania; venera@geodin.ro (V.D.); crisan@geodin.ro (C.D.)

**Keywords:** time series, causality, entropy transfer, wavelet analysis, neural networks, climate response, solar impact

## Abstract

This study addresses the causal links between external factors and the main hydro-climatic variables by using a chain of methods to unravel the complexity of the direct sun–climate link. There is a gap in the literature on the description of a complete chain in addressing the structures of direct causal links of solar activity on terrestrial variables. This is why the present study uses the extensive facilities of the application of information theory in view of recent advances in different fields. Additionally, by other methods (e.g., neural networks) we first tested the existent non-linear links of solar–terrestrial influences on the hydro-climate system. The results related to the solar impact on terrestrial phenomena are promising, which is discriminant in the space-time domain. The implications prove robust for determining the causal measure of climate variables under direct solar impact, which makes it easier to consider solar activity in climate models by appropriate parametrizations. This study found that hydro-climatic variables are sensitive to solar impact only for certain frequencies (periods) and have a coherence with the Solar Flux only for some lags of the Solar Flux (in advance).

## 1. Introduction

In the climate system, the processes that take place are due to the combination of two main factors: the solar external factor and its own internal mechanism. The complexity of this combination is difficult to quantify in space-time by deterministic–explicit or stochastic–dynamic models [1,2]. The relationship between the internal and external determinant factors in the evolution of the terrestrial climate system remains quite unknown, despite recent and increasingly sophisticated modeling.

In general, different pros and cons arguments try to bring light to different statistical methods or deterministic models about their suitability to the peculiarities of the sun–climate connection.

Even through coupled ocean-atmosphere models of general circulation, it is not always possible to adequately capture the climatic responses to solar forcing.

Thus, the sensitivity of some climate hydro-climatic processes may not represent true responses when a physical parameter does not respond linearly with the solar forcing [2]. In turn, even hydro-climatic processes have nonlinear links between them [3]. These links were highlighted using the joint entropy method. Smith [4] uses mutual information (*MI*) to calculate nonlinearity and looks at *MI* as a measure of total dependence between random variables. Goodwell et al. [5] discusses the advantages and disadvantages of applying information theory to the links of different variables in earth sciences, trying to find a measure of these links.

Multiple more-or-less sophisticated methods of detecting Granger-type causality between different factors for different fields [6,7,8] have been developed. Other investigators developed some information measures via predefined functions, copula functions [9] or relative information-generating functions as defined by Guiasu and Reischer [10] and Hao and Singh [11]. Many applications attempt to aggregate important factors to determine the evolution of natural phenomena on Earth, but few succeed. This is because the method of discerning the contribution of each factor, as from cause to effect, is not the most appropriate. It is crucial that the method of discrimination can quantify the contribution of each factor from a lot of factors that contribute to the determination of a phenomenon, and the transfer of entropy (TE) offers this desire to the full [12,13,14]. In addition, TE provides an adequate tool for relevant physical interpretations.

However, there happens to be errors of interpretation regarding TE itself [15], so this tool should be used with great care. Therefore, details that are not yet known about the physical mechanisms that govern terrestrial climate behavior remain to be found. Discrimination of the direct effects of solar activity on processes in the climate system is difficult. Solar disturbance, even quite strong, is modulated by the non-linearities existing between it and hydro-climatic factors [16]. In addition, even the internal mechanism of the atmosphere itself can produce notable climate extremes.

In addition, even the internal mechanism of the atmosphere itself can produce notable climate extremes [16,17,18]. As a secondary issue, the reassessment of the sun’s impact on the climate is also necessary due to the fact that modern satellite measurements outside the modulating space of the Earth’s atmosphere are more correct [19,20]. 

However, the situation is so complex that we have to evaluate the structure of the causal chain both internally between geophysical factors and between geophysical factors and solar activity. Depending on this situation, as well as the current state of knowledge, we decided to conduct investigations to elucidate at least partially the existing problems.

In this study, we first test the links between the external factor, described by the Solar Flux parameter 10.7 cm, and the hydro-climatic variables, whether or not they are nonlinear by a method proposed by [21,22]. If the links are nonlinear, we test complexity by appropriate measures, provided to a large extent in the paper [23]. Entropy may be looked at as a measure of complexity [24]. Of course, informational entropy in general and especially entropy transfer is regarded today as a method in advanced sciences [25]. However, entropy can be seen not only as a measure of complexity but also as a measure of causality, more precisely, as the transfer of entropy in the interrelationships of natural processes [26]. Palus and Vejmelka [27] and Goodwell and Kumar [28] consider that the conditional mutual information term is equivalent to the transfer entropy. It was also found that some source processes, recorded in hydro-meteorological time series, are more active processes compared to others counted over longer or shorter periods [5]. For other periods, the roles are changed between source and target [29]; namely, sources become passive (targets) and targets become sources (active).

The main objective of our investigation in this paper is to establish the causal chain of the solar impact with respective weights on the hydro-climatic processes using the theory of information that is able to capture aspects, which deterministic methods by inherent constraints cannot do. During our investigations, as we will see in the following, results are not always significant. Significant signals appear on certain time intervals of the analyzed time series.

Therefore, is important to find measures of process interaction in the time-frequency domain. We chose the situation of the a posteriori knowledge of the dual manifestation in time frequency of sun–climate processes through wavelet analysis [30]. How this is achieved will be seen in the paragraph below, in which we mention the characteristic intervals and consistency of the processes. It is not yet enough to know only qualitatively the details of the physical mechanisms that govern the behavior of the terrestrial climate, but also the weights of the solar impact.

Some investigations start from modeling the solar activity signal on the climate indices [31], knowing a priori partial characteristics. With the help of these models, the authors come to the interesting conclusion that suggests a causal link between solar activity and pseudo-periodicities of most climate indices. The method, although essentially linear, applied to nonlinear structures manages to capture a solar signature imprint on a significant part of the terrestrial phenomena recorded by climatic indices.

It is important to establish the existence of laws from cause to effect between external factors and the mechanisms of the geophysical environment considered by hydro-climatic indices. This is the approach we are trying to implement here by methods that implicitly capture the non-linearities of the considered links. We thus manage to discriminate between external causes and hydro-climatic factors. This is the first step towards the proper modeling of hydro-climatic processes under the impact of the external factor. 

From a theoretical point of view, things are quite advanced [26]. However, from an application point of view, some papers [5,32] used estimates of the links of active causal factors (sources) and receptors (passive) in a linear manner through representations in the space of wave numbers such as development in the Fourier series.

In general, there are pros and cons for different statistical methods as to their suitability for the nature of the connection between phenomena. That is why it is good for us to first test the nature of the link between phenomena and then conduct investigations accordingly. 

In a previous study [33], *MI* was used to estimate the nonlinear correlation coefficient between two terrestrial variables and its statistical significance was performed by generating an ensemble of 1000 surrogate data using the transition matrix of the first-order Markov chain.

As in the domain of sun influence on the hydro-climatic system with focus on the Danube basin, there are few works that address the problem from cause to effect; we try here to elucidate some aspects of the direct solar impact by applying the theory of information.

## 2. Material and Methods

### 2.1. Data

We consider the climatic variables that govern the synoptic scale through their seasonal averages. We did not consider the climate variables by our innate desire quoting [1] because, as demonstrated, the impact of the sun on the climate has distinct characteristics for the summer season compared to the winter season and even the transition seasons [34]. The present investigation refers to the seasonal, unfiltered time series from 1948 to 2000. We therefore have a statistical volume of 53 values, sufficient for entropy transfer analysis [35].

#### 2.1.1. Regional Scale

The Lower Danube Basin discharge recorded at the Orsova station (ORS_Q), located at the entrance of the Danube in Romania, was used. It represents an integrator of the upper and middle basin. Data were provided by the National Institute of Hydrology and Water Management, Bucharest, Romania. For each station, of the 15 meteorological stations considered in the Danube basin, a simple drought index (TPPI) was estimated, which was calculated by the difference between standardized temperatures and precipitation.

#### 2.1.2. Large Scale

In order to see the influence of large-scale atmospheric circulation on the variables at the regional scale, we considered the seasonal mean values of the sea level pressure field (SLP) in the sector 50° W–40° E, 30°–65° N. SLP data were available at http://rda.ucar.edu/datasets/ds010.1 (accessed on 21 March 2013) of the National Center for Atmospheric Research (NCAR). The 5-degree latitude/longitude grids, computed from the daily grids, begin in 1899 and cover the Northern Hemisphere from 15° N to the North Pole. The North Atlantic Oscillation index (NAOI) was downloaded from http://www.ldeo.columbia.edu/res/pi/NAO/ (accessed on 21 March 2013).

The Greenland–Balkan Oscillation index (GBOI), introduced by Mares et al. in 2013 [36], was calculated using the correlative analysis of the first principal component (PC1) of the Empirical Orthogonal Functions (EOFs) for the precipitation field at the 15 stations of this study with the sea level pressure (SLP) at each grid point where it was defined. Then, GBOI was calculated as differences of normalized SLP at Nuuk and Novi Sad, located in opposite sign correlation nuclei [36]. 

NAO and GBO circulation indices can capture certain aspects of climate processes on a planetary and continental scale, respectively. 

Atmospheric blocking as a variable is one of the most important phenomena whose genesis cannot be reproduced by sophisticated deterministic models. The atmospheric blocking is important by its association with extreme hydroclimatic events and is modulated by solar activity [37]. The calculation of blocking indices involves pressure differences between middle and northern latitudes. The geopotential at 500 hPa was provided by the *British Atmospheric Data Centre (BADC)* (https://badc.nerc.ac.uk/home/index.html) (accessed on 23 January 2017). Three sectors were taken into account: Atlantic–European (AE) on the domain (50° W–40° E; 35° N–65° N), Atlantic (A) defined in (50° W–0°, 35° N–65° N) and European (E) in the region (0°–40° E; 35° N–65° N). The corresponding blocking indices are AEBI, ABI and EBI, respectively. 

Climate variables were chosen at the Earth’s surface level for the simple fact that the sun–climate relationship, although evident in the higher atmospheric levels, at the Earth’s surface is not evident by the earlier investigations [34,38] and in newer ones it is partially clear [37,39].

#### 2.1.3. Solar Flux Index

For the time interval 1948–2000, the solar forcing is quantified by the solar radio flux at 10.7 cm (usually called the F10.7 index). Details on the 10.7 cm solar radio flux and its applications are given in [40]. 

We considered solar activity through Solar Flux because older and even newer studies have highlighted qualitative links between Solar Flux 10.7 with atmospheric variables such as temperature at the isobaric level of 30 hPa in the stratosphere [34]. Newer studies that have shown a good representation of total solar radiation (TSI) by Solar Flux 10.7 cm, which suggests it should be considered both in deterministic models as well as stochastic ones [41].

The Sun’s external factors impact on hydro-climatic processes at the ground surface suffers significant modulations [42]. These modulations are due to the internal mechanisms of the climate system components explained in more detail by [43] by supposition of the existence of a stratospheric jet which interacts with the atmospheric waves. These waves then transfer energy throughout the troposphere [44]. The role of solar activity in this transfer would be to increase the conversion of the baroclinic energy of the current jet to eddies with heat exchange to eddies.

### 2.2. Methods

#### 2.2.1. Preliminaries

**Figure 1:**
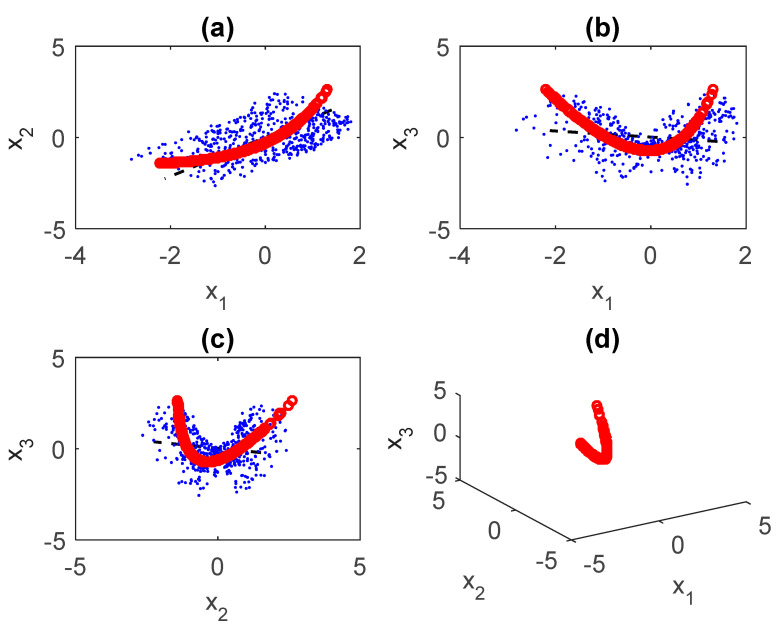
Nonlinearities between the Solar Flux (X1) and the climate variables NAOI (X2) and the Danube discharge at the Orsova station (X3) during winter (1948–2000). Plane projection: (**a**) for (X1, X2), (**b**) for (X1, X3), (**c**) for (X2, X3) and (**d**) space projection for (X1, X2, X3).

It is very important to determine whether the link between the variables describing the phenomena in question is linear or nonlinear. This analysis is carried out by appropriate methods of neural networks [21,45]. For example, as for the link between the Solar Flux and the NAOI, as well as the Danube discharge at the Orsova station—by applying neural network models to the analysis of nonlinear canonical correlation, following Hsieh and Tang [21] and Hsieh [22]—we found that it is clearly nonlinear (Figure 1).

The difference in timing is crucial in considering mechanisms to explain solar–climate links [46] and the non-linearity link test should also be applied in this case. However, more important is how we must take this difference in timing. Additionally, this is one of the purposes of this study. We are looking for such a lag in the time-frequency domain by the wavelet transform [30] that there is a coherence between the Solar Flux and the considered hydro-climatic variables. In this way we establish exactly the lag between the Solar Flux (in advance) and the terrestrial variables.

#### 2.2.2. The information Theory Elements

Applying information theory to essentially dynamic systems bring us more information in explaining what governs geophysical processes under the impact of solar external forcing. It is understandable that solar activity under its various forms should lead the phenomena of the earth. However, also between the various hydro-climatic phenomena, there must be a causal link. A huge leap was made from Granger’s simplest causal link [47] to the robust nonlinear type based on informational entropy defined by Schreiber [26].

Mutual information (*MI*) is defined by

(1) $$MI\left(X,Y\right)=H\left(X\right)+H\left(Y\right)-H\left(X,Y\right)$$

*H*-information entropy of the variables *X* and *Y* [48]

If we consider three variables, we can estimate the *MI* between two variables conditioned on a third variable. This measure is referred to as conditional mutual information and is given by Equation (2)

(2) $$MI\left(X;Y/Z\right)=H\left(X/Z\right)-H\left(X/Y,Z\right)$$

As is shown in [49], a method for quantifying the transfer of information from one variable to another was developed by [26] and then applied in many investigations [28,50,51]. 

According to Timme and Lapish [52] transfer entropy, using conditional mutual information, is given by [26]

(3) $$TE\left(X\to Y\right)=MI\left(Y_{future};X_{past}/Y_{past}\right)$$

In this study, we used Timme and Lapish’s MATLAB calculation routines [52].

Pearl shows [53] that in the last 20 years, significant progress has been made in elucidating the problem of causality in which information theory plays a very important role. In [53] it is suggested that causality, in fact, is an interaction between two phenomena of type “action (*X*)reaction (*Y*)” in which the conditions are fulfilled: one of the phenomena (effect, e.g., *Y*) undergoes structural changes [53,54] whenever the cause (*X*) appears, and the (*Y*) effect must appear so that we always have a flow (direction) of information [47] from *X* to *Y* and thus there is no confusion [52]; it also must be a direct passage, because if it is not direct, the reversal could be done by intermediate targets or for synergistic reasons [52]. The conditions mentioned are respected in the present study, with TE from Solar Flux directly leading to each of the hydro-climatic variables with a lag of at least 1 year. 

It should be noted that information theory may provide a “causal inference” [53] but is not capable of providing a “causal model” [52,53] as well as a causal inference engine. However, the theory of information with its causal inference derivative can make important contributions to the realization of predictive models.

#### 2.2.3. Wavelet Coherence

To highlight the repartition in the time and frequency domain of the coherence between two variables, we applied the wavelet analysis. Fourier transform assumes stationarity of the processes. 

A time series $\left\{X\left(t\right)\right\}$ can be analyzed by its decomposition on several components according to two parameters: the *dilation* parameter s > 0, and *translation* parameter *u*, −∞ < *u* < ∞. Such decomposition is performed through a real or complex function $\psi_{u,s}\left(t\right)$ called a wavelet and is defined as follows:

(4) $$\psi_{u,s}\left(t\right)=\frac{1}{\sqrt{s}}\psi \left(\frac{t-u}{s}\right)$$

The continuous wavelet transform (CWT) of the time series *X(t)* is defined by:

(5) $$W_{X}\left(u,s\right)=\int_{-\infty }^{+\infty }X\left(t\right)\frac{1}{\sqrt{s}}\psi^{*}\left(\frac{t-u}{s}\right)dt$$

And helps us to reconstruction the original series $\left\{X\left(t\right)\right\}$ entirely. The * sign represents the complex–conjugate of that expression.

For analysis of the covariance of two time series, the cross-wavelet spectrum (XWT) of two time series *X* and *Y* with wavelet transforms *W_X_* and *W_Y_* is obtained as:

(6) $$W_{XY}\left(s,u\right)=W_{X}\left(s,u\right)W_{Y}{}^{*}\left(s,u\right)$$

And can be considered as a measure of the correlation of the “wavelet spectra” of the two time series $X\left(t\right)$ and $Y\left(t\right)$. The cross-wavelet power, which is a measure of the common power, is calculated as $\left|W_{XY}\right|$.

A very useful tool is the wavelet coherence (WTC). Coherence is a measure of the intensity of the covariance of the two series in time-frequency space:

(7) $$R^{2}\left(s,u\right)=\frac{\left|\langle s^{-1}W_{XY}\left(s,u\right)\rangle \right|}{\langle s^{-1}{\left|W_{XX}\left(s,u\right)\right|}^{2}\rangle \langle s^{-1}{\left|W_{YY}\left(s,u\right)\right|}^{2}\rangle }$$

where 〈·〉 is a suitable smoothing operator. 

Cross-wavelet transformation and wavelet coherence provide information about the relation between two time series. Details and references are found in [30,33]. 

## 3. Results

To establish the coherence in the frequency-time domain between our data we applied wavelet coherence (WTC) analysis.

**Figure 2:**
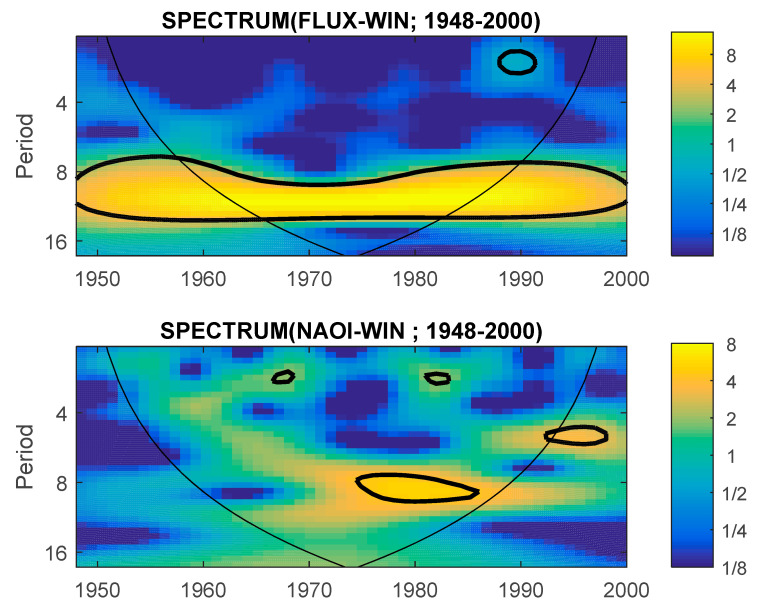
Wavelet spectrum in winter (1948–2000) for Solar Flux (top) and NAOI (bottom).

In Figure 2, as an example, the wavelet spectrum of Solar Flux (top panel) and NAOI (bottom panel) are given. The maximum of the spectra corresponds to the periods 8–12 years in both variables, with statistical significance for the whole period in case of Solar Flux, and only inside the cone of influence in case of NAOI. 

**Figure 3:**
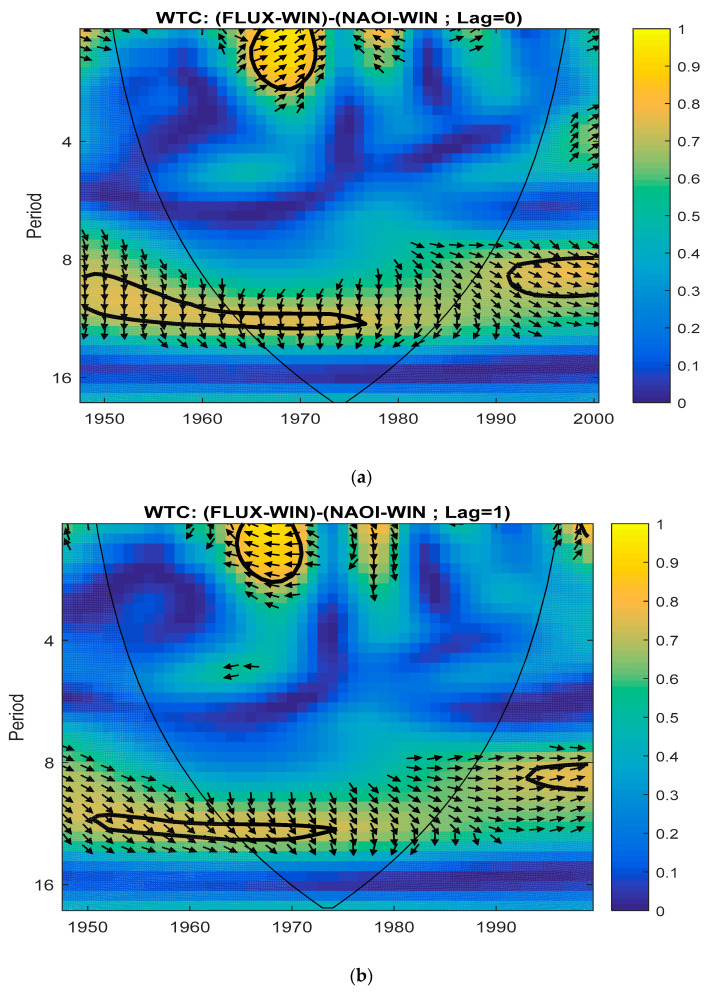

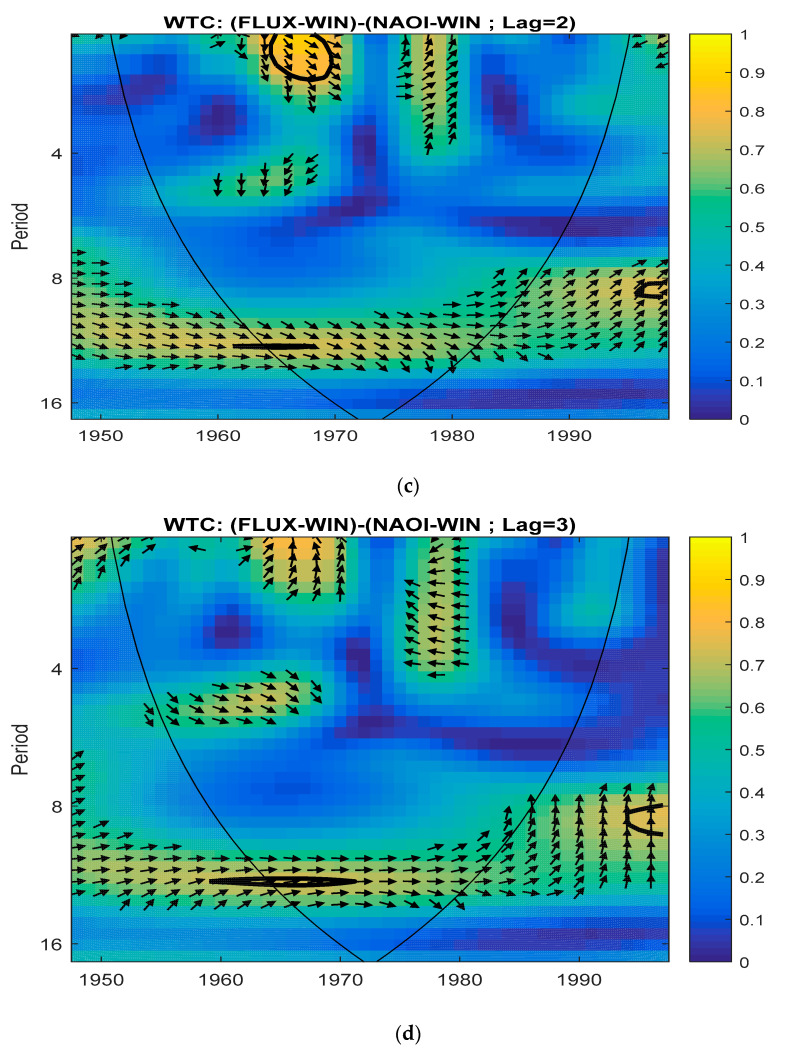
Wavelet coherence (WTC) between Solar Flux and NAOI in winter for: (**a**) Simultaneously (Lag = 0) time series (1948–2000); (**b**–**d**) Solar Flux in advance of NAOI with Lag from 1 to 3 years.

For Lag = 0, Figure 3a shows the wavelet coherence of the two phenomena simultaneously (Solar Flux and NAO) in winter. The arrows indicate phase difference. The coherence seems to be significant at least for a frequency corresponding to periods of 8–12 years, for the time interval 1948–1985.

In Figure 3b–d, the WTCs between Solar Flux and NAO are presented. The flux is taken before NAOI with Lag from 1 to 3 years (Figure 3b–d, respectively). In the area of interest (periods of 8–12 years), we notice that the arrows rotate counterclockwise with the increase of Lags, so that the phase difference becomes zero at Lag = 3. In this moment the NAO is coherent with the Solar Flux (maximum amplitude and left–right horizontal arrows). 

**Figure 4:**
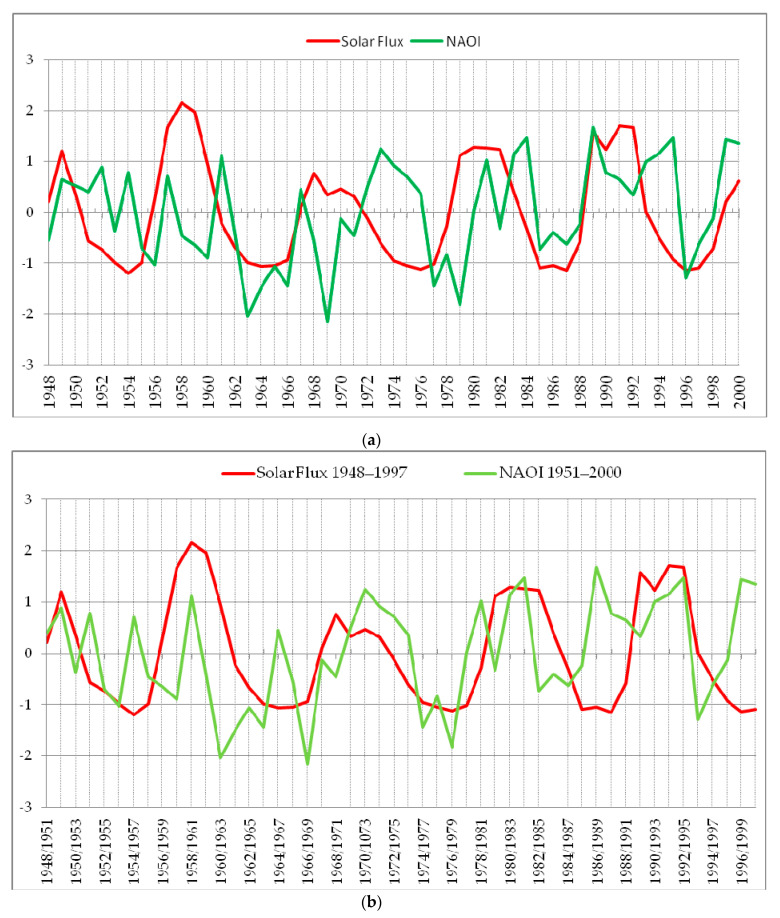
Standardized time series of Solar Flux (red) and NAOI (green) in winter for: (**a**) Lag = 0 (1948–2000) and (**b**) Lag = 3 (Solar Flux 1948–1997; NAOI 1951–2000).

A good coherence between the two time series in phase (left-right horizontal arrows) can be seen for the periods between 8 and 12 years for the first 35 years. Good coherence, in the first 35–40 years, is also displayed by the temporal evolution of the standardized time series, as can be observed in Figure 4b in comparison with Figure 4a. 

The wavelet coherence [30] was calculated for different lags from 1 to 5 years. Lag = 3 represents the moment when the two phenomena are in phase. We also found, in the case of other terrestrial variables, that they are sensitive to solar impact only for certain frequencies (periods) and these have a coherence with the Solar Flux only for certain lags of the Solar Flux (in advance).

In the following, a bidirectional analysis of the entropy transfer from Solar Flux (source) to one of the seven terrestrial variables (target) is made and the results are presented in Table 1. These results are obtained by the procedure described in [52] and the level of statistical significance *p* is displayed in parentheses. More recently, the statistical significance is also achieved through other procedures [55]. 

In [52], statistically significant level *p* for interaction estimated by TE developed the theory according to the number of states (bins) used, the statistical volume of the actual analyzed series and the statistical volume of the surrogate series. 

The authors [52] suggest the application of methods appropriate to the respective time series analyzed. In [54], a non-parametric U-test called the Mann–Whitney test is used to check the quality between two means, when fundamental assumptions are not necessary (e.g., when the two populations are not normal).

In the present study, the test for the significant level *p* (Table 1) is adapted to become more efficient using the methodologies described in [56,57,58,59].

In Table 1, the transfer entropy and the lags, for each season, from Solar Flux to the seven terrestrial variables, are presented. The analysis was achieved with a solar index taken with the lag from 1 to 5 years before terrestrial variables. 

If we consider only the cases with relative high significance *p* ≤ 0.15, we obtain the following results: in the spring season, there are four situations with entropy transfer from solar index to ABI, AEBI, TPPI and Q_ORS, for which it can be said that the Solar Flux has a direct influence on the climatic phenomena described by the corresponding indices, with lags from 1 to 4. For the summer, there is only TE from Solar Flux to AEBI for lag 4. In the fall season, it is observed that the significant causal solar impact is on EBI and Q_ORS, at lag = 3 and 2, respectively. In winter, TE appear significant for the atmospheric indices at the large-scale GBOI, NAOI and AEBI at lags from 3 to 5 years. Related to the missing values in Table 1, it should be mentioned that the TE values might also be significant for lags not tested in this study, such as lag > 5.

Therefore, if we refer only to discharge as the main hydro-climatic index, the direct causal impact on the Danube discharge can be considered significant only in the spring and fall seasons with a delay of 3 and 2 years.

## 4. Conclusions and Further Work

In this study, it was found that hydro-climatic variables are sensitive to solar impact only for certain frequencies (periods) and these have a coherence with the Solar Flux only for certain lags (in advance). 

The Danube discharge in the lower basin at Orsova station is directly sensitive (after a time interval of 2~3 years) to solar activity in the spring and fall seasons. However, the significance of TE from Solar Flux to discharge is not very high, because the Lower Danube basin discharge, in addition to solar activity, is also caused by other factors that determine its evolution.

The main conclusion is that the causal dependence of those phenomena with positive TE was proved to exist and occurs with a specific “delay”. At the same time, the interpretation of these connections is facilitated.

The selection of these causal dependencies opens the way to adequate modeling and conforming to the evolution of hydro-climatic phenomena with discriminated solar impact. 

After establishing the nature of the connection between solar and considered geophysical variables, the meaning of the link from cause to effect is of interest. Detection of the type of causal connection between natural phenomena is of great interest both for the objective explanation of mechanisms and for subsequent modeling.

Of course, other interesting ways of approaching such as that described in [31,60] illustrate that nonlinear empirical modeling can help to disentangle complex climate interactions and various factors that are perhaps even external. The disadvantage of these methods, whose robustness is obvious, is that there must be prior knowledge about the processes that take place in each system.

The original contribution of this study is that the Solar Flux F10.7 has a signature on the hydro-climatic factors focused on the Danube basin and that the impact of this signal is modulated by the internal mechanism of the atmosphere. Then, the way of obtaining these direct links from cause to effect is achieved by the robust method of entropy transfer applied after preliminary analysis. Preliminary analysis consists in optimizing the connection in the time-frequency domain with an appropriate lag to have a consistent coherence between the Solar Flux and each of the hydro-climatic variables.

The caveat of this work stems from the fact that the analysis carried out postulated the existence of a single Solar Flux source that acts on a target. A crucial yet completely unresolved problem is that multi-source interactions lead to a possible redundant impact. Important steps to elucidate this problem were made by Lizier et al. [61].

This paper is only a short contribution on how to apply the robust method of information theory (supported by wavelet analysis of nonstationary signals) to the complex links of certain geophysical phenomena under direct solar impact.

Necessarily, the next investigation that is required is to establish the links between the hydro-climatic variables themselves, in order to establish to what extent they are sources (active) or targets (passive) and for how long they have these interchangeable characteristics.

The results of the investigation presented [29] give us hope that we are heading the right way. We intend to apply this new kind of investigation to other problems in climate science.

## Figures and Tables

**Table 1:** Transfer entropy (TE) from Solar Flux to seven terrestrial variables, with the respective delays (Lags) for each season in the period 1948–2000. *p*–Statistical significance level. Values of TE for which the significance level *p* ≤ 0.15 are bolded. Missing values mean that TE < 0 for the considered lags.

	Solar Flux (Source)							
	Spring		Summer		Fall		Winter	
Terrestrial variable (Target)	TE	Lag	TE	Lag	TE	Lag	TE	Lag
GBOI	–	–	0.249 (0.61)	1	0.316 (0.37)	4	**0.425 (0.03)**	**4**
NAOI	0.258 (0.66)	4	0.287 (0.37)	2	0.270 (0.58)	5	**0.376 (0.12)**	**5**
ABI	**0.409 (0.02)**	**4**	–	–	–	–	0.079 (1.00)	5
AEBI	**0.486 (0.001)**	**1**	**0.392 (0.03)**	**4**	0.277 (0.56)	4	**0.344 (0.05)**	**3**
EBI	–	–	0.058 (0.50)	1	**0.332 (0.05)**	**3**	0.090 (0.63)	5
TPPI	**0.310 (0.06)**	**2**	0.228 (0.63)	3	0.158 (0.5)	5	–	–
Q_ORS	**0.367 (0.15)**	**3**	0.314 (0.25)	3	**0.337 (0.15)**	**2**	0.248 (0.67)	1
